# Complementary Feeding Methods, Feeding Problems, Food Neophobia, and Picky Eating among Polish Children

**DOI:** 10.3390/children11010045

**Published:** 2023-12-29

**Authors:** Agnieszka Białek-Dratwa, Oskar Kowalski

**Affiliations:** 1 Department of Human Nutrition, Department of Dietetics, Faculty of Public Health in Bytom, Medical University of Silesia in Katowice, ul. Jordana 19, 41-808 Zabrze, Poland; 2 Department of Cardiology, Congenital Heart Diseases and Electrotherapy, Silesian Center for Heart Diseases, 41-800 Zabrze, Poland

**Keywords:** BLW, baby-led weaning, neophobia, complementary feeding, children’s diet

## Abstract

Proper nutrition during the first period of life is primarily related to meeting energy needs and providing essential nutrients that ensure the infant’s normal physical and psychomotor development. Improper nutrition during this period, inadequate amounts of nutrients, inappropriate timing and manner of introduction of individual foods, can permanently alter metabolism and the course of physiological processes, increasing the risk of diseases such as obesity, allergic diseases, and cardiovascular diseases. This study aimed to verify how the method of complementary feeding influences the occurrence of food neophobia between 2 and 7 years of age, as well as to assess the different nutritional aspects resulting from the process of starting feeding other than breast milk and milk formula. In this study, 490 mothers and their children aged 2–7 years participated. The research tool was a questionnaire consisting of a child’s dietary assessment and standardized questionnaires assessing food neophobia among children: Food Neophobia Scale for Children (FNSC) and the Montreal Children’s Hospital-Pediatric Feeding (MCH-FS). In the study group of children, 238 (48.57%) had no Baby-Led Weaning Method (no BLW) method used during complementary feeding (CF), and 252 (51.42%) children used Baby-Led Weaning Method (BLW). According to the FNSC questionnaire, a high risk of food neophobia was found in 32.65% of the children studied and a medium risk in 39.80%. The medium risk of feeding problem occured in 11.63% of children, the high risk in 6.73% of children, and the highest risk in 6.94% (MCH-FS). No statistically significant differences were observed between the BLW and NoBLW groups. High risk of food neophobia occured in 1/3 of the children studied, but there was no relationship in the study group between the mode of CF (BLW/NoBLW) and the risk of food neophobia.

## 1. Introduction

Adequate nutrition during the first period of life is primarily related to meeting energy requirements, as well as providing the necessary nutrients to ensure the infant’s normal physical and psychomotor development [1]. Nutrition during this period is also important for so-called metabolic programming. Among other things, nutrition is related to the influence of environmental factors, including nutrition during critical periods of life (including early pre- and postnatal development). It influences individual development and the risk of developing diseases in later years of life. Inappropriate nutrition during this period, e.g., inadequate amounts of nutrients, inappropriate timing and manner of introduction of particular foods, can permanently alter metabolism and physiological processes, increasing the risk of diseases such as obesity, allergic diseases, and cardiovascular diseases [2,3]. Complementary feeding (CF), the introduction of foods other than breast milk or infant formula, is an important aspect in the nutrition, development, and socialization of the infant [4]. The introduction of complementary foods should begin when the infant has demonstrated the developmental skills needed to consume them—usually no earlier than 17 weeks of age (beginning at 5 months) and no later than 26 weeks of age. (beginning of 7 months of age) [1,5]. The optimal age and sequence of introduction of complementary foods is a subject of ongoing debate, and recommendations regarding these issues vary [1,5,6]. For nutritional reasons, including the need to meet the requirements for all essential nutrients, most infants require the introduction of complementary foods, in addition to breast milk and/or milk formula, from around 6 months of age. However, there is no single correct age at which complementary foods should be introduced in all infants. It depends on the characteristics and development of each infant individually [1]. According to the recommendations of The European Society for Paediatric Gastroenterology Hepatology and Nutrition (ESPGHAN), the introduction of complementary foods can begin at 17 weeks of age (i.e., beginning of 5 months) and no later than 26 weeks of age (i.e., beginning of 7 months). Beginning the introduction of complementary foods around the child’s sixth month of life is advised by the World Health Organization (WHO) [7]. Parents may be confused about when to start introducing more foods into their child’s diet due to the differences between the ES-PGHAN and WHO approaches [8]. However, for the majority of newborns, the capacity to consume solid food reaches a mature stage when they learn to sit with assistance, become neuromuscularly mature enough to control head and neck motions, and learn to feed with a spoon. This is the point at which the mouth-removal reflex, which is common in the neonatal and early infant stages and makes feeding with foods other than liquids challenging, goes away [1,6].

One of the CF methods recommended by the Polish Society of Gastroenterology, Hepatology, and Child Nutrition (PTGHiŻD) is the use of the Baby-Led Weaning (BLW) method [1]. A modified version of BLW Baby-Led Introduction to SolidS (BLISS) has been developed that specifically addresses the importance of introducing iron- and energy-rich complementary foods and avoiding foods that may pose a choking risk. The main principles of the BLISS method include feeding food in the shape of an adult’s finger, ensuring that pieces of food are long enough for the child to hold and eat at the same time, cooking food enough that it can be crushed on the palate with the tongue, and giving the child a variety of foods, including one energy-rich and one food high in fat [9]. The BLISS method also incorporates child safety rules: do not give the child foods such as peanuts, popcorn, grapes, or any food shaped shaped like a coin—the child may choke [9]. But there are no convincing arguments either for or against using the BLW method or the BLISS [1,6]. The BLW approach of introducing complementary meals is a feeding strategy that is managed by the kid. It is predicated on the parents/caregivers avoiding the spoon-feeding stage and feeding pulpy/puree foods (puree: smooth consistency, such as blended fruit and puree with lumps, such as blended fruit with cooked rice). Upon reaching approximately 6–7 months of age, when the infant can sit up without assistance, a range of hand-graspable solid foods are introduced, such as carrots, cucumber strips, apple, pear, and various pasta shapes and meat strips. It is important that these items are not too small to prevent choking hazards for the child [1,8,10].

Another stage in the CF of young children’s diet is a refusal against eating foods they are unfamiliar with. This is food neophobia, which refers to a fear/disgust/even loathing of new, unfamiliar foods, which may result in the rejection of the unfamiliar food [9]. From an evolutionary perspective, food neophobia is considered a survival mechanism [11,12]. The emergence of neophobia may also be rooted in genetic as well as environmental factors [11,13,14,15]. Most commonly, neophobia relates to the consumption of three types of food: vegetables, meat, and fruit [11,16]. Given that these three food groups are most frequently linked to toxins, germs, and allergic reactions, this pattern may have evolutionary roots [11,17]. When kids start exploring their surroundings without adult supervision—from parents, caregivers, or other adults—food neophobia reaches its apex. Therefore, when these youngsters are at a high risk of swallowing hazardous or poisonous foods, food neophobia serves as a protective mechanism [18,19,20]. Neophobia is not a lifelong aversion to trying new foods; it typically peaks between the ages of 2 and 6 [11]. It is possible to get a youngster to accept new meals and recipes by exposing them to rejected foods on a regular basis or by demonstrating their intake [20]. Item neophobia is associated with “picky” eating to some level, which is the rejection of both known and unknown meals and may be more difficult to overcome with repeated exposure to a certain item [20]. Problems with food intake are referred to by many names. Some of these categories allude to eating disorders, some to challenges with food intake of different etiologies, and some to behaviors like eating neophobia that are part of a child’s normal developmental stage [21]. Sadly, they are misunderstood by both parents and experts, leading to chaotic behaviors that are unsuitable for the illness and can have detrimental effects on one’s health or encourage unwanted behavior [21,22].

In child nutrition, it is essential to investigate how children are eating, and how they eat their meals depends on the culture of their relationship with food [23]. For example, in the DeJesus experimental study, it was observed that children preferred conventional foods, and those eating conventional foods rated those eating unconventional foods negatively [24]. Considering this aspect, children are likelier to choose conventional meal combinations, e.g., meat with potatoes instead of meat with fruit. The study by Foinant et al. supports the theory that children with high levels of food rejection show greater caution when determining whether foods are edible. They misclassify foods as inedible significantly more often than children with lower levels of food rejection [25].

Pickard et al. noted that the child’s awareness matters in meal acceptance, e.g., “if children do not have sufficient knowledge of scripted foods (e.g., eggs for breakfast) and thematically related foods (e.g., eggs with toast), we predict that they will remain uncertain about the appropriateness of the culinary scene. When an object or situation carries a potential risk, it is safer to dismiss such an event” [23]. Pickard et al.’s study posits that there exists a strong correlation between lesser understanding of theme and scripted linkages in the food domain and food rejection tendencies, namely food neophobia and food selectivity. According to the findings, kids who exhibit greater levels of food neophobia possess conceptual knowledge about food but struggle to apply that knowledge when making decisions about food [23]. 

Therefore, a feeding method such as BLW can expose the child to more foods so that the child will not be afraid of them because they will recognize them and classify them accordingly. In addition, the sensory play with food that takes place when starting to use this method makes the child more familiar with the product or dish, compared to, for example, pureed meals in which the child does not have the opportunity to get to know the ingredients. The important thing, however, seems to be that it is not the BLW supplementary feeding method itself that is important, but the amount of food and the varied form in which it is given, which will prevent the child from developing food neophobia or food selectivity in later life.

Considering the methods most commonly used during infant CF such as spoon-feeding and the BLW method, and the possibility of food neophobia already occurring during CF, this study aimed to verify how the method of CF influences the occurrence of food neophobia between 2 and 7 years of age, as well as to assess the different nutritional aspects resulting from the process of starting to provide foods other than breast milk and milk formula.

## 2. Materials and Methods

### 2.1. Study Group

The survey was carried out using a questionnaire-based, indirect survey technique using a web form (CAWI). Parents were given a very thorough instruction on how to complete the questionnaire before filling it in. Parents of children in preschool and kindergarten were able to access the questionnaire through Facebook groups for parents in particular Polish cities and areas, as well as through groups intended to facilitate communication between parents and educational institutions. The study involved selecting three kindergartens from each region and inviting them to participate. The kindergarten database, https://przedszkola.edubaza.pl/ (accessed on 15 December 2023), was accessed and closed on 15 December 2023. In turn, the selection of nurseries was based on a random selection of two nurseries from each of Poland’s voivodeships, drawn from the Ministry of Family and Social Policy’s list of nursery school registers. In these nurseries and kindergartens, groups of children older than two were chosen, and survey questionnaires were distributed using the CAWI approach to these groups of kindergartens and day nurseries. Each participant was asked to consent to the data sharing guidelines after being made aware of the survey’s objective, the fact that participation was voluntary, and their anonymity. Adult respondents to the poll were parents of preschoolers and nursery school students. According to the survey’s approved inclusion and exclusion criteria, the sample was entirely random because the CAWI method was used to conduct the survey. Due to the study participants’ increased interest in their children’s diets, there is a slight chance of inaccuracy. The study period covered the months of January–February 2023. The study was an observational retrospective study. Issues related to the infant period, such as breastfeeding, milk formula feeding, complementary feeding method, and CF time, were retrospective. On the other hand, the aspect of children’s current feeding patterns and occurring feeding difficulties such as picky eating and feeding neophobia were observational study.

### 2.2. Rationale for the Selection of the Test Group

According to current Polish law on insurance provision, a mother is entitled to maternity leave after childbirth of 20 weeks for one child, 31 weeks after the birth of twins, 33 weeks after the birth of triplets, 35 weeks for quadruplets, and 37 weeks in the case of quintuplets [26]. After the birth, the mother must compulsorily take 14 weeks of leave. She may give up the remaining six weeks of leave and return to work, provided that the remaining maternity leave is taken by the father raising the child or, for a period corresponding to the remaining maternity leave, personal care of the child is provided by the father (has social insurance) of the child who has interrupted his gainful activity in order to provide this care. After this period, both parents can take parental leave, which lasts 32 weeks if one child is born [27,28,29]. According to reports from the Social Insurance Institution (pl. Zakład Ubezpieczeń Społecznych—ZUS), more than 246,000 parents, including only 1900 men, benefited from maternity benefits for the period of parental leave in Poland from January to May 2021 [30]. Upon analyzing the parents who were polled, it was noted that mothers filled out the other questionnaires, whereas only one father had completed them. Since mothers are the ones who are usually at home with their children or in contact with an educational institution like a nursery or kindergarten and are responsible for feeding the children during this period, only mothers were retained for the study of feeding children during the period of CF and feeding during the preschool period. 

### 2.3. Exclusion and Inclusion Criteria 

The questionnaire was only made available once consent to participate in the study was obtained, and the mothers who took part in it gave their informed consent to do so. The following factors were considered in the group selection criteria: the respondents had to be of legal age, have at least one child in the nursery or preschool years, and not have any formal training in the behavioral determinants of nutrition (i.e., education or training pertaining to the treatment, education, and nutrition of children and adolescents). In Poland, children from the ages of three to seven are eligible for preschool education; however, in very specific circumstances, a child as young as five may also be enrolled. Considering the aforementioned details, the study’s inclusion requirements were as follows: the participant had to provide their consent, be the mother or legal guardian of a kid between the ages of 2 and 7, and finish filling out the questionnaire. However, in order to be excluded from the study, a child had to meet certain criteria, such as not giving their consent to participate in the study, not answering questions, being younger than two years old or older than seven years old, having a condition that affected how they should be fed, such as food allergies or intolerances, or having autism spectrum disorders. A total of 490 moms and their kids were included in the final analysis after the inclusion and exclusion criteria were taken into account.

The study was conducted in accordance with the Declaration of Helsinki and the Act on the Profession of Physician and Dentist. A positive opinion was obtained from the Bioethics Committee operating at the Silesian Medical University in Katowice to conduct a study on parents’ knowledge of young children’s nutrition (PCN/CBN/0052/KB/101/22) on 15 June 2022.

### 2.4. Research Tool

A five-part anonymous survey questionnaire served as the study tool. The first section dealt with the parent/guardian and their child and had information on delivery, current weight and height, food intolerances and allergies, as well as the parent/guardian’s age and sex, place of residence, education, and child’s sex. The WHO standard body weight of children in terms of underweight, normal weight, overweight, and obesity was assessed based on the child’s current age, weight, length/height using centile grids, and three standard deviations of body mass index (BMI) for girls and boys aged 0–3 years. For children aged 3–7 years, the developmental norms for girls and boys aged 3–18 years according to OLAF and OLAF studies were used [31,32]. For this study, the parents who completed the survey were asked to fill up the “Child Health Booklet” with details on their child’s birth, including the length of the birth, the weight at birth, and the mode of delivery (natural, planned, or unplanned cesarean).

As per the official stance of the Polish Ministry of Health, the child’s health booklet is equipped with data on the prenatal period, delivery, postpartum health status, patronage visits, preventive examinations, which encompass dental exams, history of infectious diseases, allergies, and anaphylactic reactions, radiological procedures, medical device provision, exemption from sports activities, and other pertinent information related to evaluating the child’s normal development from birth, such as weight and length measurements and growth until adulthood. All of this is achievable thanks to a standardized model. The physician, midwife, nurse, or other health care provider enters the information in the child’s health book as soon as the service is rendered; if this is not feasible, the information is completed at the subsequent visit based on the unique internal records [33].

The subsequent section of the survey centered on the feeding strategy used during infancy, considering exclusive breastfeeding, breast milk feeding, duration of breastfeeding, and the timing and technique of complementary feeding (CF) (date of CF introduction, consistency of meals during CF: puree, puree with lumps, pureed meals, meals ready for the child to eat with the fingers; products given to the child as CF and the method of expanding the infant’s diet including the use of the BLW method). 

The child’s present diet, including utensil use, food preferences, taste perception, feeding habits, and instances of food selectivity, was covered in the third section. The questionnaire was created using information about CF, such as the BLW method and food selectivity occurring during this stage of a child’s life, as well as current dietary recommendations for the group of youngest children and CF method developed by PTGHiŻD [1] based on ESPHGAN recommendations [6].

The last two sections of the questionnaire addressed the prevalence of food neophobia. The research tools used were standardized questionnaires assessing food neophobia among children: Food Neophobia Scale Children (FNSC) [34] and the Montreal Children’s Hospital-Pediatric Feeding Program [35,36]. In our study, we used the Polish version of the Montreal Children’s Hospital Feeding Scale (MCH-FS), which was appropriately translated and validated [36].

The Montreal Children’s Hospital-Pediatric Feeding Program (MCH-FS) is related to the feeding of a child from 6 months (receiving a pureed diet) to 6 years of age. It includes questions such as the following: how would you rate your child’s meal pattern? How concerned are you about your child’s meal pattern? How do you assess your child’s appetite (feeling of hunger)? At what point during a meal does your child start refusing to eat? How long do your child’s meals last (in minutes)? How do you assess your child’s behavior during mealtimes? Does your child choke, gag, spit up, or vomit at certain foods? Does your child hold food in the mouth without swallowing? Do you have to walk behind your child or distract him/her (toys, TV) to get him/her to eat? Do you have to force your child to eat or drink? How do you assess your child’s chewing (or sucking) skills? How do you assess your child’s growth (weight, height)? How does feeding your child affect your relationship with your child? How does feeding your child affect your family relationships? [35,36,37]. The MCH-FS consists of 14 items covering the following feeding characteristics: oral motility (8 and 11), oral sensory (7 and 8) and appetite (3 and 4). Other items relate to mothers’ concerns about feeding (1, 2, and 12), mealtime behavior (6 and 8), strategies used by mothers (5, 9, and 10) and family response to child feeding (13 and 14) [35]. A 7-point scale was included for each question [35,36]. Interpretation (MCH-FS) indicates from 14–45 points no risk of neophobia, 46–52 points middle difficulties, 53–58 points moderate difficulties, above 59 points most difficulties.

For the Food Neophobia Scale (FNS) questionnaire, we used the 10 items of the original FNS developed by Pliner and Hobden [34,38], backtranslated. As the FNS referred to children, we used the Children Food Neophobia Scale (FNSC). The questions in the FNSC questionnaire were worded by adding the prefix “my child”. And in the remainder of this paper, we use the abbreviation FNSC. We used the following questions: My child tries new and different foods all the time; My child does not trust new foods; If my child does not know what is in a particular food, he or she will not try it; My child likes foods from different countries; My child finds ethnic foods (minorities and ethnic groups) too strange to eat; My child tries new foods; My child is afraid to eat things that he or she has never eaten before; My child is very picky about the food we eat; My child will eat almost anything; My child would like to eat foods from other regions of Poland or other countries. In the FNSC, each item is rated on a 7-point agreement scale, from 1 = strongly disagree to 7 = strongly agree. All food neophilia statements were reversed so that the above scores indicated food neophobia. The total FNSC score was used to assess a person’s level of food neophobia and propensity to try unfamiliar foods [38]. Eating behaviors can be influenced by environmental, cultural, and social factors, so some statements were altered during translation of the scale to fit the appropriate cultural context and adapted to the Polish language [11,39].

### 2.5. Statistical Analyses

Statistica v. 13.3 (StatSoft Inc., Tulsa, OK, USA) was used for statistical analysis. The study group of children and their mothers obtained lowest and maximum values, and the range of values obtained was determined. The measured data were characterized by mean and standard deviation (X ± SD). The variables were examined using statistical tests in order to make statistical inferences. The distribution of each parameter was examined using the Shapiro–Wilk test. For statistical analysis, non-parametric tests were thus employed. Pearson’s Chi2 test was used to compare the group of children who used the BLW approach during CF with those who did not utilize the BLW method for non-parametric features and bivariate tables. The level of statistical significance adopted in the study was set at *p* ≤ 0.05. The value of the Cramer’s V coefficient was calculated for the adopted level of significance *p* ≤ 0.05. The Cramer’s V coefficient takes values between 0 and +1 (inclusive), where the closer the result is to 0, the weaker the relationship between the studied characteristics, and the closer it is to 1, the stronger the relationship between the studied characteristics. We used this coefficient to assess the association between the FNSC and MCH-FS food neophobia risk scores and the mothers’ subjective opinion of their children being picky eaters.

## 3. Results

### 3.1. Characteristics of the Study Population

This study involved 490 mothers and 490 of their children. Table 1 shows the characteristics of the study group of mothers and their children. The mean age among the study mothers was 33.36 ± 4.93 years, while the mean age of the study children was 4.18 ± 1.6 years. The mean gestational age at which the study children were born was 39.00 ± 1.99 weeks of gestation. In this study, we took into account the average length of exclusive breastfeeding (3.86 ± 0.8 months); exclusive breastfeeding according to the WHO definition means not giving milk formula to the baby because the baby consumes only breast milk. We also assessed the average length of breast milk feeding, which was 10.9 ± 5.89 months in the study group of children. 

Table 2 shows the results characterizing the place of residence of the mothers and children, the education of the mothers, as well as the sex of the children studied, the current age of the child and, based on the current data presented, body weight and height were related to the BMI centile grids. On this basis, the current prevalence of underweight, normal weight, overweight, and obesity among the study children was established. The study also included a division into two groups, among which mothers declared whether BLW (BLW) or no BLW (NoBLW) was used during CF. In the studied group of children, 238 (48.57%) did not have the BLW method applied during CF, and 252 (51.42%) children had the BLW method applied. Considering the place of residence of the children surveyed, 31.09% of the NoBLW children and 42.46% of the BLW children lived in a city of more than 100,000. The mothers of BLW children were mostly characterized by higher education (78.97%), and the same was true for the group of mothers of NoBLW children (66.81%). The gender distribution of the children in the entire study group was as follows—48.98% boys and 51.02% girls. Analyzing the current age of the children in the whole study group, we found that 20.00% were 2-year-olds, 21.63% 3-year-olds, 17.14% 4-year-olds, 14.69% 5-year-olds, 14.69% 6-year-olds, and 11.84% 7-year-olds. Analyzing body weight, we found that most children were characterized by normal weight (69.18%), 23.67% were underweight, while 5.10% in the whole study group were overweight and 2.04% obese.

Table 3 considers the feeding method immediately after birth and during the CF period. Among the BLW children studied, mothers were far more likely to feed exclusively with breast milk until 6 months of age, and as many as 55.56% of mothers did so. This compared to only 29.83% of mothers of NoBLW children. Mothers exclusively breastfeeding up to 6 months were more likely to use the BLW method than mothers breastfeeding for shorter periods. Breast milk-fed children over 13–24 months and over 24 months were more likely to be fed the BLW method than children fed breast milk for a short time (up to 6 months). The BLW method was more often used in children where CF was introduced after 6 months of age than in children where CF was introduced between 4 and 6 months of age. Children fed puree and puree with lumps were less likely to use the BLW method in CF. Among mothers of BLW children, the length of breastmilk feeding was also significantly longer: 19.05% of mothers fed beyond 24 months and 29.37% from 13-24 months, while mothers of NoBLW children only 8.40% fed beyond 24 months and 17.75% from 13-24 months. The length of exclusive breastmilk feeding was analyzed; in the NoBLW group, it lasted on average 3.06 months, while in the BLW group it lasted 4.25 months (*p* = 0.0001), and the length of total breastmilk feeding among NoBLW children lasted 8.6 months, while among BLW children it lasted 13.3 months (*p* = 0.0000).

The majority of NoBLW children had CF introduced between 4 and 6 months of age 59.24%, while BLW children were more likely to have CF introduced after 6 months of age (66.67%). Considering the start of CF and the introduction of puree and puree with lumps into the daily diet, BLW children had them introduced in 74.60% and 75.00%, respectively. In contrast, children in the NoBLW group 96.22% had puree introduced and 65.55% had puree with lumps. Most children in both the BLW and NoBLW groups had other vegetables such as pumpkin, carrots, and parsley introduced as the first food (47.90% NoBLW; 54.76% BLW). Green vegetables such as broccoli and courgette were also frequently introduced to a large extent—18.07% of NoBLW children and 21.43% of BLW children started CF with these foods. Considering the essence of self-feeding in the BLW method, it was taken into account how often the child was spoon-fed: 26.98% of BLW children ate completely or mostly on their own, indicating full use of the BLW method. In contrast, among the NoBLW children, 45.80% were mostly or entirely spoon-fed and 52.52% were half spoon-fed by an adult. 

The study also took into account current recommendations that the child decides how much to eat and the parent decides what the child eats [1,11]. Among NoBLW children, 33.61% decided what to eat, while in the BLW group as many as 72.62% did. With respect to the amount of food eaten, in the BLW group as many as 93.65% decided how much they eat, while in the NoBLW group 80.67% did. The question of the occurrence of difficulties in introducing new foods to the child during CF was also considered. In both study groups, mothers declared the absence of problems (NoBLW 68.91%; BLW 70.24%).

Statistically significant differences between the NoBLW and BLW baby groups were the method of delivery (*p* = 0.00108), the length of time the baby was fed exclusively with breast milk (*p* < 0.001), the length of time the baby was fed with breast milk (*p* = 0.00002), the time of starting CF (*p* < 0.001), the introduction of puree (*p* < 0.001) and puree with lumps (*p* = 0.00261), spoon-feeding during CF (*p* < 0.001), letting the baby decide what to eat (*p* < 0.001) and how much to eat (*p* = 0.00008). Mothers of children born by natural delivery or unplanned cesarean section were more likely to use the BLW method than mothers of children born by planned cesarean section. 

The following feeding problems occurred among children during CF: in both study groups it was observed that the most common problem in this age group was spitting food out of the mouth—corresponding NoBLW 60.08% and BLW 68.65%—*p* = 0.04764. After first defining for parents the differences between gagging, choking, and choking with medical intervention, the incidence of gagging was NoBLW 23.53% and BLW 40.87%—*p* = 0.00004, respectively. Considering choking occurred in 5.46% in the NoBLW group and 8.33% in the BLW group, medical intervention was needed in one child in the BLW group. An airway that is completely blocked is referred to as choking. A baby that is choking will not make much noise since their airway is blocked. The infant may turn blue, grasp at their throat, or show signs of extreme distress. When someone is choking, a caregiver must typically step in and push the food out of their airway. It is common for an infant learning to eat to gag reflexively. When food gets stuck in the back of a baby’s mouth, they will gag, cough, and splutter to get the food back in the front of their mouth. Unlike choking, gagging typically makes noise [40].

The incidence of the vomiting reflex, choking and choked, and needed medical attention was not different in the two study groups (Table 4).

Table 5 shows the results on current cutlery skills during mealtimes. Most of the children surveyed used a spoon and fork. However, there were differences between the average age of starting to use cutlery. BLW children significantly started using cutlery earlier than NoBLW children. The mean age of acquisition of the ability to use a spoon while eating meals was 17.8 months in the NoBLW group, 15.0 months in the BLW group (*p* = 0.00001), while the NoBLW fork was 20.0 months, BLW 17.3 months (*p* < 0.001), and fork and knife at one time in the NoBLW group was 3.8 years, BLW 3.5 years (*p* = 0.00275).

Considering the main aim of the study and the issues related to food selectivity, the parent’s perception of feeding was analyzed and it was found that most children in both study groups had an appetite and eat almost everything (Table 6). However, it was observed that if the child did not want to eat, mothers of NoBLW children had to force the child to eat, while mothers of BLW children did not force the child to eat. According to their mothers, 23.47% of the children in the study group were picky eaters, 3.67% of the children ate only dishes with a certain consistency, and 5.31% of the children only ate products with selected tastes. The study observed that there were no significantly significant differences in children’s perceptions of parental eating (*p* = 0.16666), children eating a particular texture of meals (*p* = 0.07682), and children eating a particular taste of meals (*p* = 0.94616). In contrast, there was a statistically significant difference in children’s food choosiness. Children in the NoBLW group were more likely to be food choosers according to their mother than children in the BLW group (*p* = 0.04766).

### 3.2. Interpretations of Food Neophobia Based on Standardized Questionnaires

**Figure 1:**
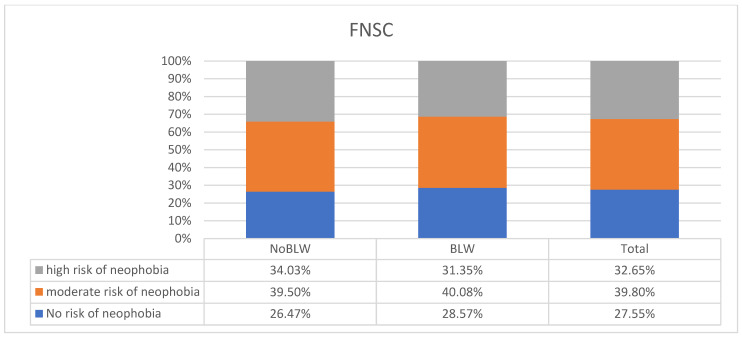
Assessment of the risk of food neophobia using the FNSC among the children surveyed with the BLW method (N = 490).

**Figure 2:**
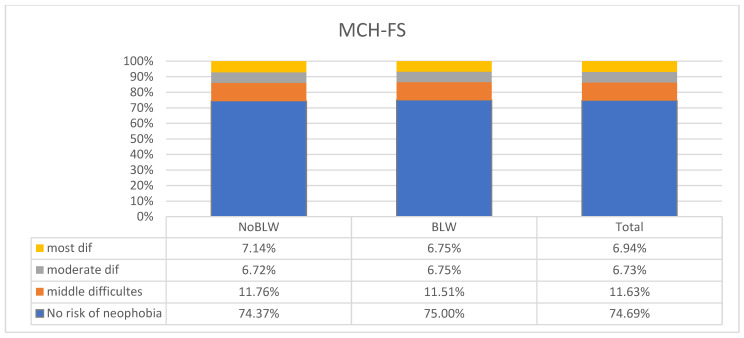
Assessment of the risk of feeding problems using the MCH-FS among the children surveyed with the BLW method (N = 490).

The study analyzed a group of children for the risk of food neophobia. Two questionnaires, FNSC and MCH-FS, were used (Figure 1 and Figure 2). According to the FNSC questionnaire, a high risk of feeding difficulties was present in 32.65% of the children studied and a medium risk in 39.80% of the children. No statistically significant differences were observed between the BLW and NoBLW groups in feeding difficulties. By analyzing the results obtained from the MCH-FS questionnaire, we determined that the risk of the food feeding problem including food neophobia is lower than according to the FNSC. The medium risk of food neophobia was found in 11.63% of children, the high risk in 6.73% of children, and the highest risk in 6.94%. No statistically significant differences were observed between the BLW and NoBLW groups and the prevalence of feeding neophobia.

**Figure 3:**
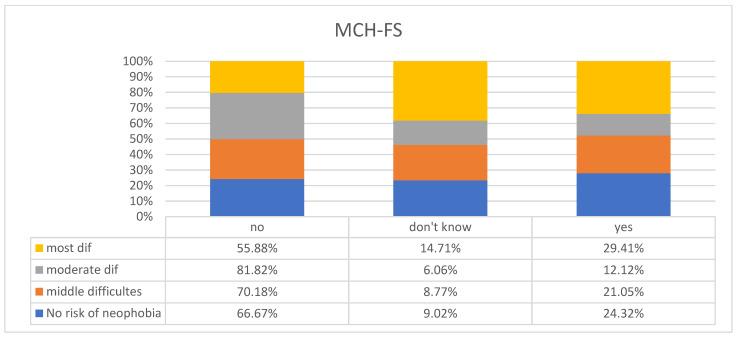
Mothers’ subjective assessment of their children being picky eaters vs. feeding problems including food neophobia with questionnaire (MCH-FS).

**Figure 4:**
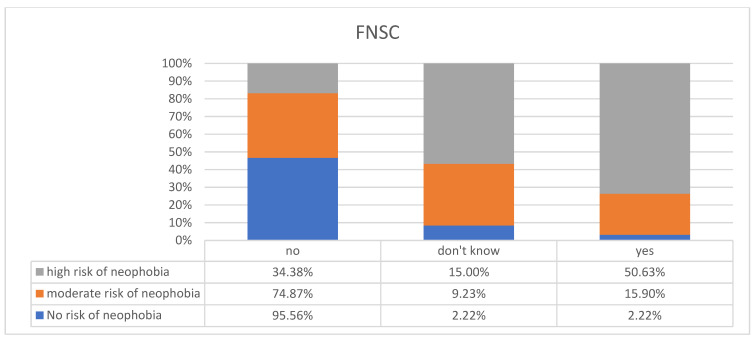
Mothers’ subjective assessment of their children being picky eaters vs. risk of food neophobia with questionnaire (FNSC).

We also analyzed the mothers’ own feelings towards the possibility of food neophobia among their children and obtained the following results. Among children who might be at risk of feeding problems (according to the MCH-FS scale), only 29.41% of mothers considered their children to be picky eaters, 14.71% of mothers found it difficult to judge, and 55.88% declared that their children were not picky eaters (*p* < 0.001 Crammer’s V = 0.3769551) (Figure 3). Considering the FNSC, we observed that among mothers whose children on the FNSC scale might be at risk of neophobia, as many as 50.63% considered their child to be a picky eater (*p* < 0.001 V Crammer = 0.4362327) (Figure 4).

## 4. Discussion

Another technique for introducing CF to babies is BLW. Most babies can chew, sit up without assistance, and take food into their mouths by the time they are 6–7 months old, suggesting that a gradual switch from purees to finger foods may not be required at this point [41]. CF using the BLW method is becoming increasingly popular. Therefore, in this study, we considered the use of the BLW method as a CF method and evaluated different aspects of infant and toddler feeding. We also wanted to verify to what extent the BLW method influences the presence or absence of food neophobia in later childhood.

It seems important to note that there is no standardized definition of BLW feeding universally, so parents may interpret this feeding method differently using the full BLW, partial BLW, or unconscious BLW model [42,43]. The full BLW model refers to the child self-feeding at 90% of meals with minimized puree feeding [44], partial BLW involves the use of spoon-feeding by an adult and self-feeding by the child, while unconscious BLW involves parents being unaware of the BLW method but allowing the child to self-feed. However, the definition is quite problematic, due to the subjective assessment of feeding by the mothers surveyed, the assessment of portion size, and the playfulness with food that takes place when using the BLW method. 

Different studies have used different divisions that consider whether the child was fed using the BLW method or not. In our study, we used the method of dividing children into a BLW group and a NoBLW group based on the mother’s description of the extent to which the child was fed: whether it was mostly spoon-fed—in which case it was qualified for the NoBLW group—or mostly self-feed—in which case it was qualified for the BLW group. In our own study, 48.57% of the children surveyed had no BLW method used during CF and 51.42% of the children had used the BLW method. In another own study, among 320 mothers participating in the study, 75.0% used the BLW method [45], and in another study, among the mothers surveyed, 63.93% mothers used the BLW method and 34.36% mothers did not use the BLW method [43]. In the study by Pearse, which analyzed the diet of infants aged 6–12 months—BLW infants were spoon-fed for ≤10% of the time and given puree for ≤10% of the time, as declared by their parents. Of the study group, 60 were classified as spoon-fed and 36 as BLW-fed [46]. In the study by Rowan, 26 infants had CF introduced by the full BLW method, while 45 used the traditional approach, i.e., spoon-feeding [47]. Based on infant feeding at 6–7 months of age, participants were divided into groups in the study by XiaoXi Fu: TSF (mostly or all adults spoon-fed), partial BLW (half adults spoon-fed, half self-feeding) or full BLW (mostly or all self-feeding). In the study group, full BLW was used in 18% of children, partial BLW in 11%, and 72% had no BLW method used [48]. Given these results, it is important to distinguish and clearly define in the study what BLW is, the percentage the child is spoon-fed, and the percentage the child self-feeds. 

In our own research, BLW-fed children remain breastfed longer, and this is also confirmed by our previous studies [8,43,45] and the studies by Martí-Solson [49], Moore [50], Brown [44], Rowan [51], Cameron [52], Morison [53], and D’Andrea [54]. Furthermore, studies among children with CF using the BLW method have shown that periods of choking occur more frequently at 6 months of age and become less frequent after 8 months [40,55]. These findings suggest that choking during feeding is related to functional chewing and swallowing ability, which improves with age [56]. In the current self-reported study, gagging occurred in 23.53% of the NoBLW group and 40.87% of the BLW group, respectively. In contrast, choking occurred in 5.46% in the NoBLW group and 8.33% in the BLW group, while medical intervention was needed in one child in the BLW group. The results of the current study confirm that gagging occurs more frequently among children using the BLW method. In our earlier research on the implementation of the BLW approach, we considered the incidence of choking, gagging, and situations where medical assistance was required. A total of 6.94% of children fed using the BLW method and 5.42% of children fed by spoon experienced choking. Nevertheless, gagging was far more common than choking; in fact, 51.90% of the children fed using the BLW approach experienced gagging, compared to just 29.10% in the spoon-fed group [43]. Brown’s study found that 13.6% of the 155 babies who had choked at least once had done so in the tight BLW group, 11.9% in the loose BLW strategy, and 11.6% in the traditional group. The sample’s overall low rate of one or more choking incidents (13.6%) did not substantially change based on the weaning group, the amount of spoon-feeding, or the usage of purees. As a result, babies who received standard spoon-feeding, a loose BLW method, or a rigid BLW approach had the same chance of choking [40]. According to a study by Cameron SL, 30% of kids fed BLW experienced at least one episode of choking following the introduction of solid food [57]. A study by Quintiliano-Scarpelli D et al. found that suffocation, choking, and gaging were quite common, occurring in 78.2%, 28.4%, and 3.1% of cases, respectively [58]. Choking incidences were significantly lower in our sample than in previous studies.

Picky eating can affect the intake and quality of children’s diets, which can consequently negatively affect their growth as well as their development [59,60,61]. Studies on the correlation between picky eating and disease risk have shown that there is an increased risk of depression, eating disorders, emotional, and behavioral problems in these children [59,62,63]. Research suggests that picky eating behavior may be related to genetic determinants and environmental factors, but the cause of the development of picky eating in early childhood is largely unknown [58,61,63]. 

A meta-analysis by Cole [59] looked for correlations of picky eating and food neophobia among children up to 30 months of age, and it found no association between breastfeeding and picky eating. The relationship between breastfeeding length and food neophobia was not confirmed—studies showed mixed results; one study showed a negative association with food dissatisfaction, while another showed no association with food neophobia. No association was found between breastfeeding and observed infant food acceptance/rejection [64]. Brown, on the other hand, showed that CF by BLW was negatively associated with picky eating in children aged 18–24 months, but this study used the Child Eating Behavior Questionnaire (CEBQ) [65], whereas in this study we used FNSC and MCH-FS and used a study group aged 2–7 years. 

In our study, we did not include an assessment of the energy consumed and nutrients in children’s daily nutrition. We believe that the specificity of nutrition at this age is very heterogeneous and difficult to estimate properly. In children fed with breast milk, it is difficult to assess the amount of milk drunk and the intake of nutrients. At the same time, it should be taken into account that some children may vomit, urinate, or spit out the meal they have eaten, and here another difficulty arises in estimating the amount of food consumed. In addition, children fed by the BLW method may drop some of the food on the floor or play with it, which makes the standard methods of estimating portions used in studies for adults erroneous. However, according to a study by Taylor, there were no differences in energy intake between the BLISS (modified BLW method) group and the control group [66]. Given the concerns about the BLW approach including inadequate energy and nutrient supply, there are very little data on this topic. The randomized study by Taylor and Fangupo specifically addressed these issues, in the same cohort, but has methodological weaknesses that call the results into question [42].

Zhao observed that neophobic children are more likely to reject foods commonly considered healthy, including vegetables, fruit, various types of salads, fish, or even poultry. Consequently, their diet may be low in protein, magnesium, and monounsaturated fatty acids, and overly rich in monounsaturated, unobtrusive, and seemingly safe carbohydrates such as bread and flour dishes [67]. 

In a study by Kozieł-Kozakowska of children aged 2.5–7 years [68], neophobic children had a diet poorer in vegetables compared to children with low levels of neophobia, resulting in deficiencies in meeting the standard for vitamin C and thiamine. Adolescents with high neophobia scores were far less likely to eat eggs, cooked or raw veggies, or legumes; in contrast, they were more likely to consume snacks and sweets, and they were also more likely to eat these items in between meals. On the other hand, no correlation between children’s anthropometric characteristics and neophobia levels—high or low—was discovered.

In our study, a high risk of food neophobia was found in 32.65% of the children studied and a medium risk in 39.80% of the children, taking into account the FNSC questionnaire. Analyzing the results obtained with the MCH-FS questionnaire, we found that the risk of food neophobia is lower than according to the FNSC. The medium risk of food neophobia is found in 11.63% of children, the high risk in 6.73% of children, and the highest risk in 6.94%. In both questionnaires, no correlation was observed between the use of BLW and NoBLW during CF. 

However, given the FNSC and MCH-FS scales used in the study and the study’s own observations of the mothers of the children surveyed, there are significant differences between mothers’ perceptions of the risk of food neophobia. However, it must be borne in mind that the questionnaire is a screening test that talks about the risk of food neophobia and is not a diagnosis. At the same time, mothers who are not qualified to assess their children’s diets may misjudge their children’s diets.

## 5. Strengths and Limitations of the Study

Our survey has limitations that must be considered when interpreting the results. The absence of educational level differentiation within the study group (mostly higher education) is a weakness of the research. As a retrospective study, ours may have contributed to the incidence of false memory effect for the specifics of CF and infancy, particularly among mothers of older children, specifically those between the ages of 5 and 7. However, we must point out that a limitation of the study is the mothers’ subjective assessment of the introduction to CF using the BLW method. We verified the use of the BLW method with several questions and based on these, we classified the child into the BLW or NoBLW group ourselves, but it must be borne in mind that this type of study may contain a memory error.

Furthermore, the survey was carried out utilizing the CAWI method, which has been frequently criticized for lacking insight into the data collection process. Nevertheless, it is important to remember that this kind of data collection is generally acknowledged and practical for gathering substantial amounts of information from groups that are frequently hard to reach. The study’s group size is a benefit because, up until now, most research on the use of the BLW approach has been conducted in smaller groups. There are not many studies on this topic, particularly in Poland. Furthermore, no cross-sectional study has examined the connection between the prevalence of food neophobia in preschoolers and the application of the BLW technique during CF. 

## 6. Conclusions

BLW children were fed exclusively with breast milk for longer and fed with breast milk in general than children in the NoBLW group. The majority of NoBLW children had CF introduced between 4–6 months of age 59.24%, while children in the BLW group were more likely to have CF introduced after 6 months of age (66.67%). The results of the current study confirm that gagging is more common among children using the BLW method. BLW children start using cutlery earlier with both spoon, fork, and knife and fork than NoBLW children. A high risk of food neophobia is present in one third of the children studied, but there is no correlation in the group studied between the method of CF (BLW/NoBLW) and the risk of food neophobia.

## Figures and Tables

**Table 1:** Characteristics of the study group of mothers and their children (N = 490).

		Average	Standard Deviation	Min–Max
Characteristics of the group of mothers surveyed	age (years)	33.36	±4.93	20–60
Characteristics of the study group of children	gestational age and child birth (gestational week)	39.00	±1.99	29–42
	length of breast-milk-only feeding (in months)	3.86	±0.8	0–6
	length of breast milk feeding (in months)	10.9	±5.89	0–33
	current age (years)	4.18	±1.6	2–7
	current weight (kg)	17.73	±5.21	9–48
	current body height (cm)	107.19	±13.43	80–150
	current BMI (kg/m^2^)	15.20	±1.79	10–24.4

**Table 2:** Characteristics of the studied group of mothers and children regarding the use of the BLW method during their child’s complementary feeding (N = 490).

	Not Using BLW (NoBLW) (n = 238)		Use of BLW (n = 252)		Total (n = 490)		*p*-Value
	n	%	n	%	n	%	
Place of residence:							*p* = 0.02175
City ≥ 100,000 inhabitants	74	31.09	107	42.46	181	36.94	
City of 50–100,000 inhabitants	56	23.53	63	25.00	119	24.29	
City of 10–50,000 inhabitants	39	16.39	33	13.10	72	14.69	
City ≤ 10,000 inhabitants	18	7.56	8	3.17	26	5.31	
village	51	21.43	41	16.27	92	18.78	
Mother’s education:							*p* = 0.00410
Higher	159	66.81	199	78.97	358	73.06	
Medium	60	25.21	50	19.84	110	22.45	
Professional	9	3.78	1	0.40	10	2.04	
Basic	10	4.20	2	0.79	12	2.45	
Gender of the child:							*p* = 0.42328
Boy	121	50.84	119	47.22	240	48.98	
Girl	117	49.16	133	52.78	250	51.02	
Current age of the child [in years]:							*p* = 0.00068
2	35	14.71	63	25.00	98	20.00	
3	56	23.53	50	19.84	106	21.63	
4	33	13.87	51	20.24	84	17.14	
5	33	13.87	39	15.48	72	14.69	
6	48	20.17	24	9.52	72	14.69	
7	33	13.87	24	9.92	58	11.84	
Child’s current weight [BMI percentile]:							*p* = 0.47349
Underweight	52	21.85	64	25.40	116	23.67	
Normal weight	171	71.85	168	66.67	339	69.18	
Overweight	12	5.04	13	5.16	25	5.10	
Obesity	3	1.26	7	2.78	10	2.04	

**Table 3:** Feeding regimen in the period immediately after birth and during complementary feeding in the study group of children including the use of the BLW method during complementary feeding (N = 490).

	Not Using BLW (NoBLW) (n = 238)		Use of BLW (n = 252)		Total (n = 490)		*p*-Value
	n	%	n	%	n	%	
Method of delivery:							*p* = 0.00108
Naturally	121	50.84	145	57.54	266	54.29	
Unplanned cesarean section	49	20.59	69	27.38	118	24.08	
Planned cesarean section	68	28.57	38	15.08	106	21.63	
For how long the baby was fed ONLY breast milk							*p* < 0.001
Not fed with breast milk	20	8.40	18	7.14	38	7.76	
Less than 1 month	71	29.83	41	16.27	112	22.86	
Up to 2 months	15	6.30	10	3.97	25	5.1	
Up to 3 months	10	4.20	10	3.97	20	4.08	
Up to 4 months	18	7.56	9	3.57	27	5.51	
Up to 5 months	20	8.40	21	8.33	41	8.37	
Up to 6 months	71	29.83	140	55.56	211	43.06	
I do not remember	13	5.46	3	1.19	16	3.72	
Breast milk feeding time:							*p* = 0.00002
Not fed with breast milk	10	4.2	8	3.17	18	3.67	
Less than 1 month	42	17.65	20	7.94	62	12.65	
1–2 months	27	11.34	22	8.73	49	10.00	
3–4 months	22	9.24	20	7.94	42	8.57	
5–6 months	22	9.24	8	3.17	30	6.12	
6–12 months	45	18.91	39	15.48	84	17.14	
13–24 months	42	17.65	74	29.37	116	23.67	
Over 24 months	20	8.4	48	19.05	68	13.88	
I continue to feed	6	2.52	12	4.76	18	3.67	
I do not remember	2	0.84	1	0.40	3	0.61	
Timing of the CF:							*p* < 0.001
Before 4 months of age	15	6.30	0	0	15	3.06	
Between 4–6 months of age	141	59.24	83	32.94	224	45.71	
After the baby is 6 months old	78	32.77	168	66.67	246	50.20	
I do not remember	4	1.68	1	0.40	5	1.02	
Introducing puree * during a child’s complementary feeding:							*p* < 0.001
Yes	229	96.22	188	74.60	417	85.10	
Not	5	2.10	62	24.60	67	13.67	
I do not remember	4	1.68	2	0.79	6	1.22	
Introduction of puree with lumps ** during child’s complementary feeding							*p* = 0.00261
Yes	156	65.55	189	75.00	345	70.41	
Not	62	26.05	58	23.02	120	24.49	
I do not remember	20	8.4	5	1.98	25	5.10	
The group of food products with which CF was started							*p* = 0.11954
Green vegetables	43	18.07	54	21.43	97	19.80	
Potatoes	25	10.50	22	8.73	47	9.59	
Other vegetables	114	47.90	138	54.76	252	51.43	
Domestic fruit (apple, pear, plum)	34	14.29	15	5.95	49	10.00	
Citrus fruits	0	0.00	2	0.79	2	0.41	
Berries	1	0.42	2	0.79	3	0.61	
White meat	1	0.42	1	0.40	2	0.41	
Eggs	2	0.84	0	0.00	2	0.41	
Gluten-free products	11	4.62	7	2.78	18	3.67	
Cereal products containing gluten	6	2.52	3	1.19	9	1.84	
All at once	0	0.00	7	2.78	7	1.43	
I did not pay attention to it	1	0.42	1	0.40	2	0.41	
Fed/supplemented by spoon during complementary feeding:							*p* < 0.001
The child ate completely or mostly independently	4	1.68	68	26.98	72	14.69	
Baby fully or mostly spoon-fed by an adult	109	45.80	19	7.54	128	26.12	
Baby half fed by an adult with a spoon, half eating independently	125	52.52	165	65.48	290	59.18	
When expanding the diet, did you allow your child to decide for himself/herself what to eat?							*p* < 0.001
Yes	80	33.61	183	72.62	263	53.67	
Sometimes	101	42.44	59	23.41	160	32.65	
Not	57	23.95	10	3.97	67	13.67	
When expanding the diet, did you let your child decide how much to eat?							*p* = 0.00008
Yes	192	80.67	236	93.65	428	87.35	
Sometimes	32	13.45	12	4.76	44	8.98	
Not	14	5.88	4	1.59	18	3.67	
Occurrence of difficulties in introducing new foods to the child during complementary feeding:							*p* = 0.85869
There was no	164	68.91	177	70.24	341	69.59	
Yes there were	57	23.95	61	24.21	118	24.08	
I do not remember	16	6.72	14	5.56	30	6.12	

* puree—smooth consistency, e.g., blended fruit. ** puree with lumps, e.g., blended fruit with cooked rice.

**Table 4:** Occurrence of problems during complementary feeding considering the use of the BLW method (N = 490).

Problems during Complementary Feeding	Not Using BLW (NoBLW) (n = 238)		Use of BLW (n = 252)		Total (n = 490)		*p*-Value
	n	%	n	%	n	%	
Vomiting reflex	64	26.89	77	30.56	141	28.77	*p* = 0.37047
Spitting food out of mouth	143	60.08	173	68.65	316	64.49	*p* = 0.04764
Gagging	56	23.53	103	40.87	159	32.45	*p* = 0.00004
Choking	13	5.46	21	8.33	34	6.97	*p* = 0.21129
Choked and needed medical attention	0	0	1	0.40	1	0.20	*p* = 0.33064

**Table 5:** Current ability to use cutlery among children surveyed N = 490.

	Not Using BLW (NoBLW) (n = 238)		Use of BLW (n = 252)		Summary (n = 490)		*p*-Value
	n	%	n	%	n	%	
Using a spoon	232	97.48	251	99.60	483	98.57	*p* = 0.04766
Using a fork	231	97.06	250	99.21	481	98.16	*p* = 0.07682
Using a fork and knife at the same time	106	44.54	113	44.84	219	44.69	*p* = 0.94616
Average age of entry into service:							
By teaspoon (in months)	17.8		15.0		16.4		*p* = 0.00001
Fork (in months)	20.9		17.3		19.1		*p* < 0.001
With a fork and knife at the same time (in years)	3.8		3.4		3.6		*p* = 0.00275

**Table 6:** Current difficulties in children’s daily nutrition among surveyed children N = 490.

	Not Using BLW (NoBLW) (n = 238)		Use of BLW (n = 252)		Total (n = 490)		*p*-Value
	n	%	n	%	n	%	
Parent’s perception of food:							*p* = 0.16666
The child often does not want to eat and I have to encourage/force him to do so	73	30.67	44	17.46	117	23.88	
The child has an appetite and eats almost everything he is given	129	54.20	133	52.78	262	53.47	
The child does not want to eat, but I do not force him to	25	10.50	68	26.98	93	18.98	
I did not pay attention to it	11	4.62	7	2.78	18	3.67	
Do you think your child is an picky eater?							*p* = 0.04766
Not	152	63.87	178	70.63	330	67.35	
I do not know	24	10.08	21	8.33	45	9.18	
Yes	62	26.05	53	21.03	115	23.47	
Does your child only eat dishes with a certain consistency (e.g., mush, lumps, small pieces)?							*p* = 0.07682
No, can and does eat meals with different consistencies	222	93.28	244	96.83	466	95.1	
I do not know/difficult to say	4	1.68	2	2.38	6	1.22	
Yes	12	5.04	6	0.79	18	3.67	
Does the child only eat dishes with a specific taste (e.g., only sweet, only salty)?							*p* = 0.94616
Yes, he only consumes products with the flavor of his choice.	16	6.72	10	3.97	26	5.31	
I do not know/difficult to say	12	5.04	13	5.16	25	5.10	
No, it consumes products from different taste groups.	210	88.24	229	90.87	439	89.59	

## Data Availability

The data presented in this study are available on request from the corresponding author. The data are not publicly available due to restrictions that apply to the availability of these data.
